# An integrated omics analysis reveals the gene expression profiles of maize, castor bean, and rapeseed for seed oil biosynthesis

**DOI:** 10.1186/s12870-022-03495-y

**Published:** 2022-03-29

**Authors:** Nian Liu, Jing Liu, Shihang Fan, Hongfang Liu, Xue-Rong Zhou, Wei Hua, Ming Zheng

**Affiliations:** 1 Oil Crops Research Institute of the Chinese Academy of Agricultural Sciences, Key Laboratory of Biology and Genetic Improvement of Oil Crops, Ministry of Agriculture and Rural Affairs, Wuhan, China; 2 CSIRO Agriculture & Food, Canberra, ACT Australia

**Keywords:** Seed oil biosynthesis, Maize, Castor bean, Rapeseed, Transcriptome analyses, Lipid-related genes, Carbohydrate-related genes

## Abstract

**Background:**

Seed storage lipids are valuable for human diet and for the sustainable development of mankind. In recent decades, many lipid metabolism genes and pathways have been identified, but the molecular mechanisms that underlie differences in seed oil biosynthesis in species with developed embryo and endosperm are not fully understood.

**Results:**

We performed comparative genome and transcriptome analyses of castor bean and rapeseed, which have high seed oil contents, and maize, which has a low seed oil content. These results revealed the molecular underpinnings of the low seed oil content in maize. First of all, transcriptome analyses showed that more than 61% of the lipid- and carbohydrate-related genes were regulated in castor bean and rapeseed, but only 20.1% of the lipid-related genes and 22.5% of the carbohydrate-related genes were regulated in maize. Then, compared to castor bean and rapeseed, fewer lipid biosynthesis genes but more lipid metabolism genes were regulated in the maize embryo. More importantly, most maize genes encoding lipid-related transcription factors, triacylglycerol (TAG) biosynthetic enzymes, pentose phosphate pathway (PPP) and Calvin Cycle proteins were not regulated during seed oil synthesis, despite the presence of many homologs in the maize genome. Additionally, we observed differential regulation of vital oil biosynthetic enzymes and extremely high expression levels of oil biosynthetic genes in castor bean, which were consistent with the rapid accumulation of oil in castor bean developing seeds.

**Conclusions:**

Compared to high-oil seeds (castor bean and rapeseed), less oil biosynthetic genes were regulated during the seed development in low-oil seed (maize). These results shed light on molecular mechanisms of lipid biosynthesis in maize, castor bean, and rapeseed. They can provide information on key target genes that may be useful for future experimental manipulation of oil production in oil plants.

**Supplementary Information:**

The online version contains supplementary material available at 10.1186/s12870-022-03495-y.

## Background

Plant storage lipids are renewable and economically valuable resources; they are important for human diet and for use as biodiesel [1, 2]. There is a pressing need for enhancing the seed oil content (SOC) of oil crops to meet the sharply increasing demand for plant oil consumption. Lipid biosynthesis is a complex biological process which is precisely regulated by multiple genes [3]. In general, fatty acids (FAs) are de novo synthesized in the plastid from acetyl-coenzyme A (acetyl-CoA) precursors, condensing into long-chain acyls by numerous enzymes such as acetyl-CoA carboxylase (ACCase) [4–6], malonyl-CoA:ACP malonyltransferase (MCMT), β-ketoacyl-ACP synthase (KAS) complex [7, 8], hydroxyacyl-ACP dehydrase (HAD), enoyl-ACP reductase (ENR), β-ketoacyl-ACP reductase (KAR) [9], and acyl carrier protein (ACP). The products of FA synthesized with chain length of C16 or C18 can be unsaturated by stearoyl-ACP desaturase (SAD). Both saturated and unsaturated FAs are released from acyl-ACP by acyl-ACP thioesterase (FAT) [10] and exported to cytosol. The resulting FAs pools are then transported to the endoplasmic reticulum (ER) for triacylglycerol (TAG) assembly by enzymes such as glycerol 3-phosphate acyltransferase (GPAT) [11, 12], lysophosphatidic acid acyltransferase (LPAAT) [13], phosphatidate phosphatase (PP) [14], diacylglycerol acyltransferase (DGAT) [9, 15–17], and phospholipid:diacylglycerol acyltransferase (PDAT) [18, 19]. Finally, the mature TAGs are transferred and stored in subcellular structures called oil bodies or oleosomes. Oleosin, caleosin, and steroleosin have been shown to be indispensable for oil body formation and can regulate SOC [3, 20]. Many transcription factors (TFs) also participate in seed development and nutrient accumulation. TFs such as WRINKLED1 (WRI1), LEAFY COTYLEDON1/2 (LEC1/2), ABSCISIC ACID INSENSITIVE3 (ABI3), FUSCA3 (FUS3), PICKLE (PKL), and VP1/ABI3-LIKE (VAL) have been proved to regulate SOC in multiple species such as Arabidopsis, rapeseed, soybean, castor bean, and others [14, 21–24].

Lipid biosynthesis requires substrates and energy from carbon metabolism. There is strong evidence showing that glycolysis provides acetyl-CoA, dihydroxyacetone phosphate, ATP, and NADH for lipid biosynthesis [25–27], whereas the pentose phosphate pathway (PPP) mainly produces the reductant NAPDH that is needed for fatty acid synthesis [27]. It has been shown that many carbohydrate-related genes impact lipid accumulation. For example, pyruvate kinase (PK) catalyzes the production of pyruvate and ATP in glycolysis, and the loss of function of *PK* in Arabidopsis drastically reduced FA content [28, 29]. Phosphoenolpyruvate carboxylase (PEPC) catalyzes the production of oxaloacetate (OAA) from phosphoenolpyruvate (PEP), and RNA interference of *PEPC* increased the oil content of cotton [30].

For decades, studies on seed oil biosynthesis have been mainly focused on comparisons within species. The genetic populations with different oil contents have been analyzed to find out the key limiting factors that regulate the seed oil content within species in soybean, rapeseed, sesame, peanuts, sunflower, maize, castor bean, and so on [31–37]. Extensive technical approaches, for instance, quantitative trait locus (QTL) analyses [38–40], genome-wide association study (GWAS) [41–43], transcriptomics, lipidomics, metabolomics and fluxomics analysis [32, 35, 36, 44–46], had been applied to uncover the molecular mechanism of seed oil biosynthesis. Nevertheless, studies on lipid metabolism within species have been carried out for more than 40 years, plenty of the vital factors that control the seed oil biosynthesis remain poorly understood. Thus, to break the bottleneck of lipid metabolism researches and seek for some new alternative genes for the improvement of oil content for plant oil industry, it is critical to explore the conserved and diverse lipid metabolic pathways across multiple species.

With the rapid development of sequencing technologies in recent years, several comparative transcriptome analyses have been performed to explore the molecular mechanisms that underlie differences in seed oil biosynthesis between different species. The transcriptomes of four developing oilseeds (*Ricinus communis*, *Brassica napus*, *Euonymus alatus*, and *Tropaeolum majus*) were sequenced based on deep expressed sequence tags (ESTs) and some conserved lipid-related modules were found [47]. Comparative genomic analyses were conducted on three low-oil grasses (*Sorghum bicolor*, *Setaria italica*, and *Oryza sativa*) and four high-oil dicots (*Glycine max*, *Gossypium raimondii*, *R. communis*, and *Arabidopsis thaliana)* to investigate the mechanisms of their differences in seed oil accumulation [48]. Zhang et al. [49] performed an integrated omics analysis to investigate acyl lipid metabolism genes and carbon metabolism genes in soybean, rapeseed, Arabidopsis, and sesame seeds. Different plant species store lipids in different organs or tissues, therefore, it is important to understand the mechanism difference in oil accumulation from different tissues. Most oil plants are exalbuminous seeds and store lipids in the embryo [50, 51], but some albuminous seeds such as castor bean and *Jatropha curcas* store lipids in the endosperm [8, 14, 52]. Additionally, plants that have developed pericarps, such as the oil palm, can accumulate lipids in the mesocarp [53]. To date, the molecular mechanisms that underlie differences in lipid accumulation among different plant species remain unclear. Comparisons between species have mainly focused on whole seeds, but differences in seed oil biosynthesis at the level of the specific lipid storage tissues have not been addressed.

Maize is one of the most important worldwide food crops and vital source of vegetable oil. To meet the increasing demand of vegetable oil, underlying molecular mechanism of oil biosynthesis in maize is of great importance. Maize kernel consists of developed embryo and endosperm, and the oil mainly stored in embryo. Maize has relatively large seeds but unfortunately the oil content of maize kernels is extremely low, which is approximately 4%–5% of the seed dry weight [54]. Nevertheless, castor bean, which had developed embryo and endosperm as well, had high oil content and accumulated plenty of oil in the endosperm. To date, inter-species comparative transcriptome analyses were carried out among limited species, and the molecular mechanism of differential oil biosynthesis between maize and other oil plants remains obscure. To uncover the molecular mechanisms responsible for differences in lipid accumulation in albuminous seeds with high- and low-oil content. Embryos of maize (*Zea mays*) and rapeseed (*B. napus*) (which is a reference of oil plants for the oil biosynthesis analysis in embryo) and endosperm tissues of castor bean (*R. communis*) were collected from developing seeds at different developmental stages for transcriptome analyses. Although the gene copy numbers for the individual genes are different among these three species, clear difference in regulation of lipid biosynthetic pathways was observed. Higher number of the lipid- and carbohydrate-related genes were regulated in castor bean and rapeseed than in maize, and the expression levels of oil biosynthetic genes were highest in castor bean.

## Results

### The copy numbers of most lipid- and carbohydrate-related genes were higher in maize than castor bean

**Fig. 1:**
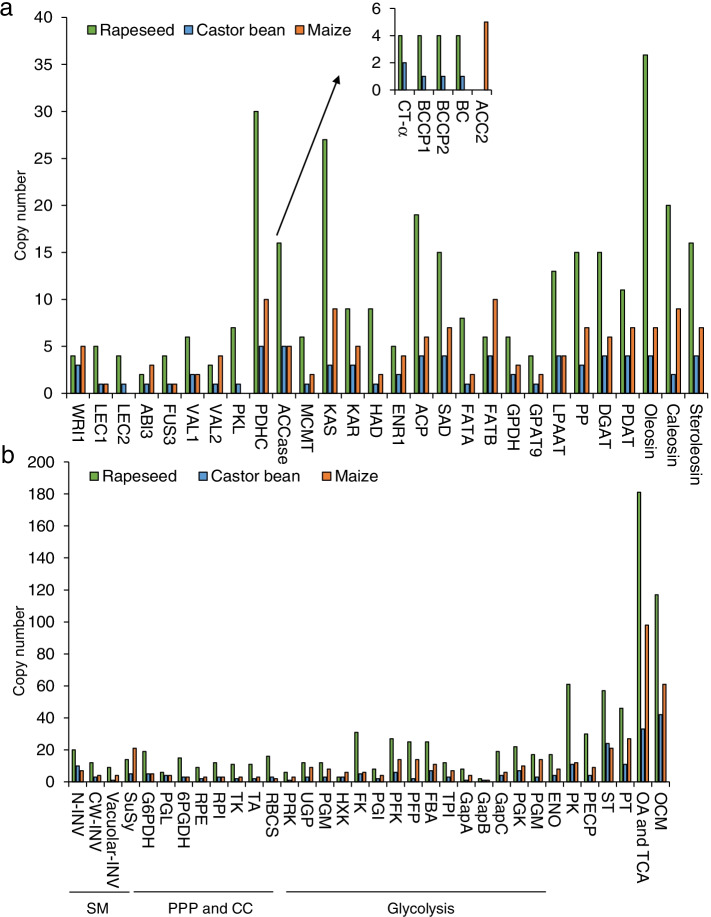
Relative copy number variation analyses of lipid- and carbohydrate-related genes in three species. a, lipid-related genes including FA biosynthesis, TAG assembly, oil body formation, and lipid-related TFs. b, carbohydrate-related genes that participated in different carbon metabolism pathways. SM, sucrose metabolism; PPP and CC, pentose phosphate pathway and calvin cycle; ST, sugar transporters; PT, plastid transporters; OA and TCA, organic acid and TCA; OCA, other carbohydrate metabolism

To eliminate the effect of species ploidy, the relative copy numbers of key regulators that participate in seed oil biosynthesis were analyzed in these three species. Copy numbers of genes that encoding proteins involving in FA biosynthesis, TAG assembly, and oil body formation were higher in rapeseed than in castor bean and maize (Fig. 1a and Table S1). This result probably reflects the fact that rapeseed is an allopolyploid and contains duplicates of most genes [55]. All lipid biosynthesis genes had more copies in maize than in castor bean except for ACCase, the key rate-limiting enzyme of FA biosynthesis (Fig. 1a and Table S1). It has been well-known that ACCase contains the homomeric ACC2 and heteromeric ACCase complex composed of CT-α, CT-β, BCCP, and BC subunits. Our findings revealed that the castor bean and rapeseed genomes contained the heteromeric ACCase but not the homomeric ACC2, whereas the maize genome only contained homomeric ACC2 (Fig. 1a and Table S1). In addition to different copy numbers of lipid biosynthesis genes, the three species also had different copy numbers of genes encoding oil biosynthesis regulators. All lipid-related TFs had fewer copy numbers in castor bean than in rapeseed (Fig. 1a and Table S1). The relative copy numbers of lipid-related TF genes in maize were inconsistent: maize had the greatest numbers of *WRI1*, *ABI3*, and *VAL2* genes among three species, but the lowest copy numbers of *LEC1* and *FUS3*. More importantly, *LEC2* and *PKL* genes were not present in the maize genome (Fig. 1a and Table S1). In addition, rapeseed had the highest copy numbers of carbohydrate-related genes that functioned in sucrose metabolism, glycolysis, the PPP, and the Calvin Cycle, followed by maize and castor bean (Fig. 1b and Table S1). These findings revealed that the copy numbers of most lipid- and carbohydrate-related genes were highest in rapeseed, lower in maize, and lowest in castor bean.

### Less lipid-related pathways were regulated in maize embryo

**Fig. 2:**
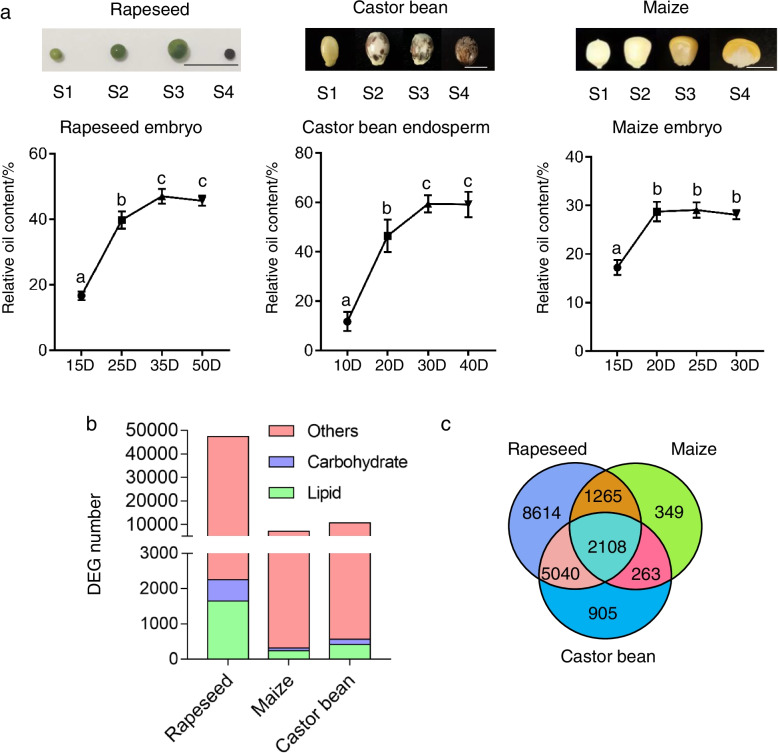
Transcriptome analyses of maize, castor bean, and rapeseed during the seed development. **a** the seed phenotypes and seed oil content of rapeseed embryo, castor bean endosperm, and maize embryo at four developmental stages (S1-S4). **b** numbers differential expressed gene (DEG) of maize, castor bean, and rapeseed during seed development. **c**, venn diagrams of DEGs in three species, all genes were compared to Arabidopsis to find the homologous genes. Scale bars, 1 cm. Values are means with SD. One-way ANOVA was used to calculate significance

Rapeseed embryo, castor bean endosperm, and maize embryo tissues were collected at four developmental stages (S1 to S4) for SOC and transcriptome analysis (Fig. 2a). The results showed that SOC increased as seed development progressed and reached its highest level at stage 3 in maize, castor bean, and rapeseed (Fig. 2a). After transcriptome sequencing and data processing, relative gene expression levels at stages 2, 3, and 4 were compared to that at stage 1 to identify the differentially expressed genes (DEGs). In total, 47 408, 10 708, and 7144 genes were differentially expressed over the course of seed development in maize, castor bean, and rapeseed, respectively (Fig. 2b). Moreover, lipid- and carbohydrate-related genes accounted for 3.5% and 1.3% of all DEGs in the rapeseed embryo, and similar numbers were found in the endosperm of castor bean or the embryo of maize (Fig. 2b). In contrast, when the number of lipid- and carbohydrate-related DEGs was compared with the number of lipid- and carbohydrate-related genes in the entire genomes, 61.8% (937/1516) of the lipid-related genes and 68.0% (607/892) of the carbohydrate-related genes in rapeseed were regulated in embryo; 61.2% (224/366) of the lipid-related genes and 61.7% (142/230) of the carbohydrate-related genes in castor bean were regulated in endosperm (Table 1). However, only 20.1% (112/558) of the lipid-related genes and 22.5% (82/365) of the carbohydrate-related genes in maize were regulated in the embryo (Table 1). To further investigate the regulation of every homologous lipid- and carbohydrate-related gene in these three species, we calculated a regulation ratio for each homologous gene by dividing the number of differentially expressed gene copies in a species by the total number of gene copies in the species. The comparison of the regulated ratio between three species showed that most genes had a higher regulation ratio in castor bean and rapeseed than in maize (Fig. S1). We therefore concluded that fewer genes involved in seed oil accumulation were regulated during seed development in maize.

**Table 1:** The number of total and differential expressed lipid-related and carbohydrate-related genes in maize, castor bean, and rapeseed

	All lipid-related genes	Differential expressed lipid-related genes	All carbohydrate-related genes	Differential expressed carbohydrate-related genes
Rapeseed	1516	937 (61.8%)	892	607 (68.0%)
Maize	558	112 (20.1%)	365	82 (22.5%)
Castor bean	366	224 (61.2%)	230	142 (61.7%)

We next performed Gene Ontology (GO) analysis to identify differentially regulated pathways between three species during seed oil accumulation. GO terms enriched in the rapeseed DEGs were mainly related to photosynthesis, response to stresses such as cold, water deprivation and abscisic acid, and lipid metabolic processes (Table S2). Castor bean DEGs were mainly enriched in GO terms related to protein biosynthesis and modification, lipid metabolic processes, and DNA replication (Table S2). In contrast, maize DEGs were enriched in GO terms such as regulation of transcription, DNA-templated, and spermine biosynthetic process (Table S2). Thus, more lipid related pathways were regulated in castor bean and rapeseed than in maize during seed development.

Next, homologous genes were identified in maize, castor bean, and rapeseed, and 2108 genes were found to be conserved among these species. Furthermore, there were 5040 dicot-specific genes (shared by castor bean and rapeseed) and 1256 embryo-specific genes (shared by rapeseed and maize) (Fig. 2c). Enriched GO terms in these 2108 conserved genes included acetyl-CoA biosynthetic process from pyruvate, long-chain fatty acid biosynthetic process, carbohydrate metabolic process, and acyl-CoA metabolic process (Table S2). Those 5040 dicot-specific genes were enriched in GO terms such as negative regulation of fatty acid biosynthetic process, lipid catabolic process, and lipid oxidation, while those 1265 embryo-specific genes were enriched in GO terms including response to salt stress, response to auxin, regulation of jasmonic acid-mediated signaling pathway, and ethylene biosynthetic process (Table S2). Therefore, we speculated that oil biosynthesis and carbon partitioning were differentially regulated between maize, castor bean, and rapeseed seeds and that fewer oil biosynthesis pathways were regulated in maize than in castor bean and rapeseed.

### More lipid metabolic, but less lipid biosynthetic genes were regulated in maize embryo during the seed development

**Fig. 3:**
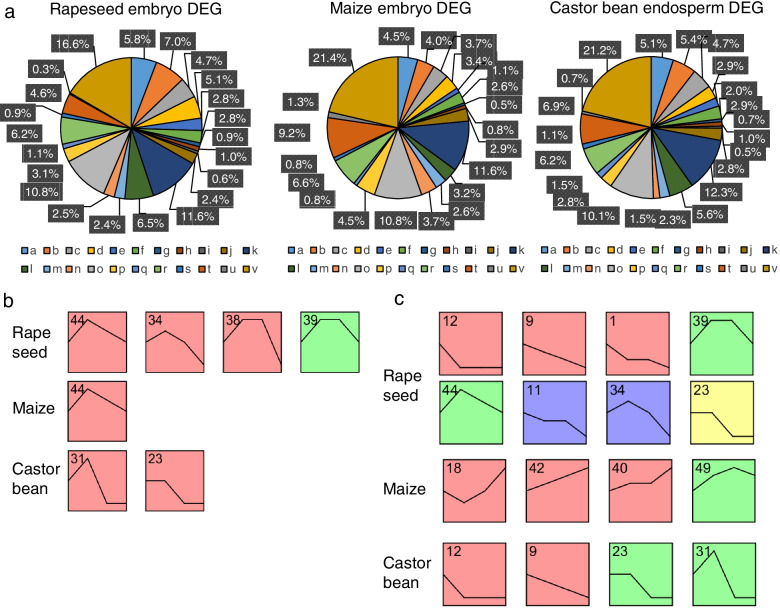
Lipid metabolism pathways and the gene expression patterns of LDEGs of maize, castor bean, and rapeseed. **a**, the proportion of maize, castor bean, and rapeseed LDEGs in different lipid metabolism categories. a, plastidial fatty acid synthesis; b, TAG synthesis; c, plastidial glycerolipid, galactolipid and sulfolipid synthesis; d, eukaryotic phospholipid synthesis; e, beta-oxidation; f, TAG degradation; g, mitochondrial fatty acid and lipoic acid synthesis; h, mitochondrial phospholipid synthesis; i, lipid trafficking; j, sphingolipid synthesis; k, lipid signaling; l, miscellaneous: lipid related; m, fatty acid elongation and cuticular wax synthesis; n, cutin synthesis; o, cuticular wax synthesis; p, aliphatic suberin synthesis; q, aromatic suberin synthesis; r, phospholipase; s, lipid acylhydrolase; t, GDSL; u, galactolipid degradation; v, lipase. b and c, the expression pattern clusters of lipid biosynthetic (**b**) and lipid metabolic (**c**) DEGs in three species

Subsequently, we focused on the gene expression profiles of lipid-related genes that have been reported to regulate lipid biosynthesis, and classified these genes into different categories based on lipid-related metabolic pathways [3]. Comparison of lipid-related DEGs (LDEGs) between species revealed that fewer lipid biosynthesis genes such as plastidial fatty acid synthesis and TAG synthesis genes were regulated in maize than in castor bean and rapeseed (Fig. 3a). By contrast, more lipid metabolism genes such as those encoding cutin synthesis, aliphatic suberin synthesis, GDSL, galactolipid degradation, and lipase were regulated in maize (Fig. 3a).

All DEGs in three species could be grouped into different clusters based on their gene expression patterns (Fig. S2). Nevertheless, rapeseed lipid biosynthesis genes were clustered into four expression profiles; these genes reached their highest expression levels at stage 2 (Fig. 3b). Likewise, maize and castor bean lipid biosynthesis genes were clustered into one and two expression profiles, respectively, and their highest expression levels were also appeared at stage 2 (Fig. 3b). Maize lipid metabolism genes were clustered into four expression profiles and showed increasing expression trends during seed development (Fig. 3c). By contrast, lipid metabolism genes in the four expression profiles of castor bean tended to decrease in expression (Fig. 3c). Similarly, genes in five out of eight rapeseed expression profiles also tended to decrease in expression during development, except profiles 34, 39 and 44, whose genes showed a temporary increase in expression at stage 2 or stage 3 (Fig. 3c). Thus, we concluded that fewer lipid biosynthesis genes but more lipid metabolism genes were regulated in the maize embryo during seed oil accumulation.

### Most TFs and genes that associated with TAG and FA biosynthesis were differentially regulated between maize, castor bean and rapeseed

**Fig. 4:**
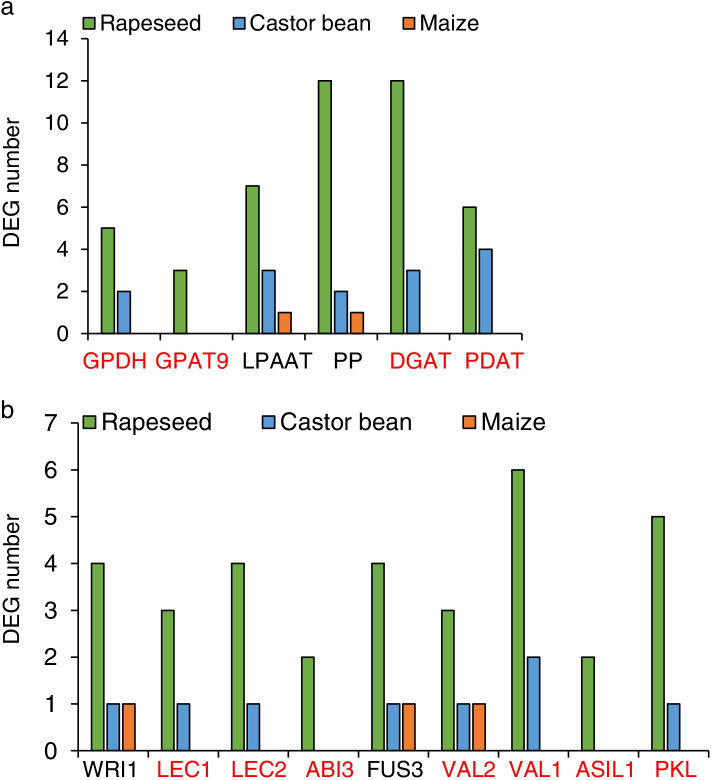
Numbers of differential expressed genes that participated in TAG biosynthesis (**a**) and vital lipid-related TFs (**b**) of maize, castor bean, and rapeseed during seed development

Comparison of differentially expressed lipid related genes between species showed that more genes involved in FA biosynthesis and oil body formation were differentially expressed in castor bean and rapeseed than in maize (Fig. S3), even though these genes had higher copy numbers in maize than in castor bean (Fig. 1a). Interestingly, most key TAG biosynthesis enzymes (e.g., glyceraldehyde-3-phosphate dehydrogenase (GPDH), GPAT9, DGAT, and PDAT) showed no regulation during maize embryo development (Fig. 4a). Similarly, key TFs that participate in lipid biosynthesis (e.g., LEC1/2, ABI3, VAL1/2, ASIL1, and PKL) were not regulated in the maize embryo (Fig. 4b). Therefore, we concluded that lack of regulation of lipid biosynthetic enzymes, especially key TAG assembly enzymes and lipid-related TFs, might lead to the low abundance of oil in maize embryo.

**Fig. 5:**
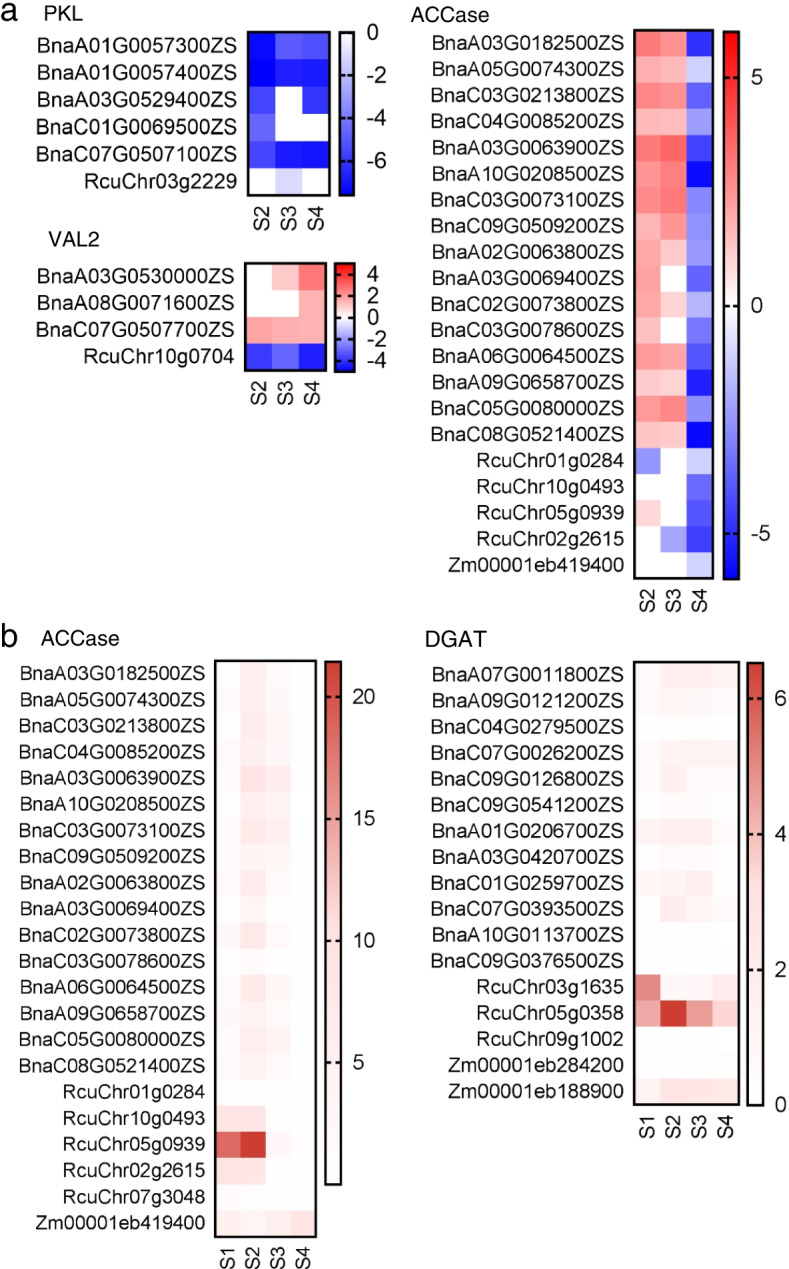
The regulation of lipid biosynthetic genes during the seed development between three oilseeds. **a** the fold changes of genes that encoding ACCase and two lipid-related TFs: PKL and VAL2. **b** the relative gene expression levels of some lipid biosynthesis genes: ACCase and DGAT. To evaluate the relative gene expression levels, the FPKMs were used for the calculation, and the average FPKM of all genes was introduced as the internal standard to evaluate the relative gene expression levels in every species

In addition to LDEG numbers, gene expression levels also differed between these species and may have led to differences in seed oil biosynthesis. There were significant differences in the fold changes of many lipid-related genes between maize, castor bean, and rapeseed. According to the comparison of fold changes between these species, the key lipid biosynthesis regulator genes *PKL* and *VAL2* showed opposite regulation patterns between castor bean and rapeseed at the four developmental stages, and they were not regulated in maize (Fig. 5a). The rate-limiting FA biosynthesis ACCase enzymes were up-regulated at stage 2 and stage 3 in rapeseed, but ACCase homologs were down-regulated or not regulated in castor bean and maize at stage 2 and stage 3 according to the fold changes (Fig. 5a). Similarly, numerous lipid-related genes (e.g., *FUS3*, *WRI1*, *LEC1/2*, *PDCH*, *GPDHC*, and *DGAT*) were also differentially regulated in maize, castor bean, and rapeseed according to the comparison of fold changes (Fig. S4). To compare the gene expression levels of lipid-related genes in three species, the average FPKM of all expressed genes was introduced to homogenize the relative gene expression levels in each species. We found that most lipid-related genes had higher relative expression levels in castor bean than in rapeseed and maize (only homologous *ACCase* and *DGAT* genes were shown), a finding that might account for the rapid accumulation of oil in castor bean endosperm (Fig. 5b).

**Fig. 6:**
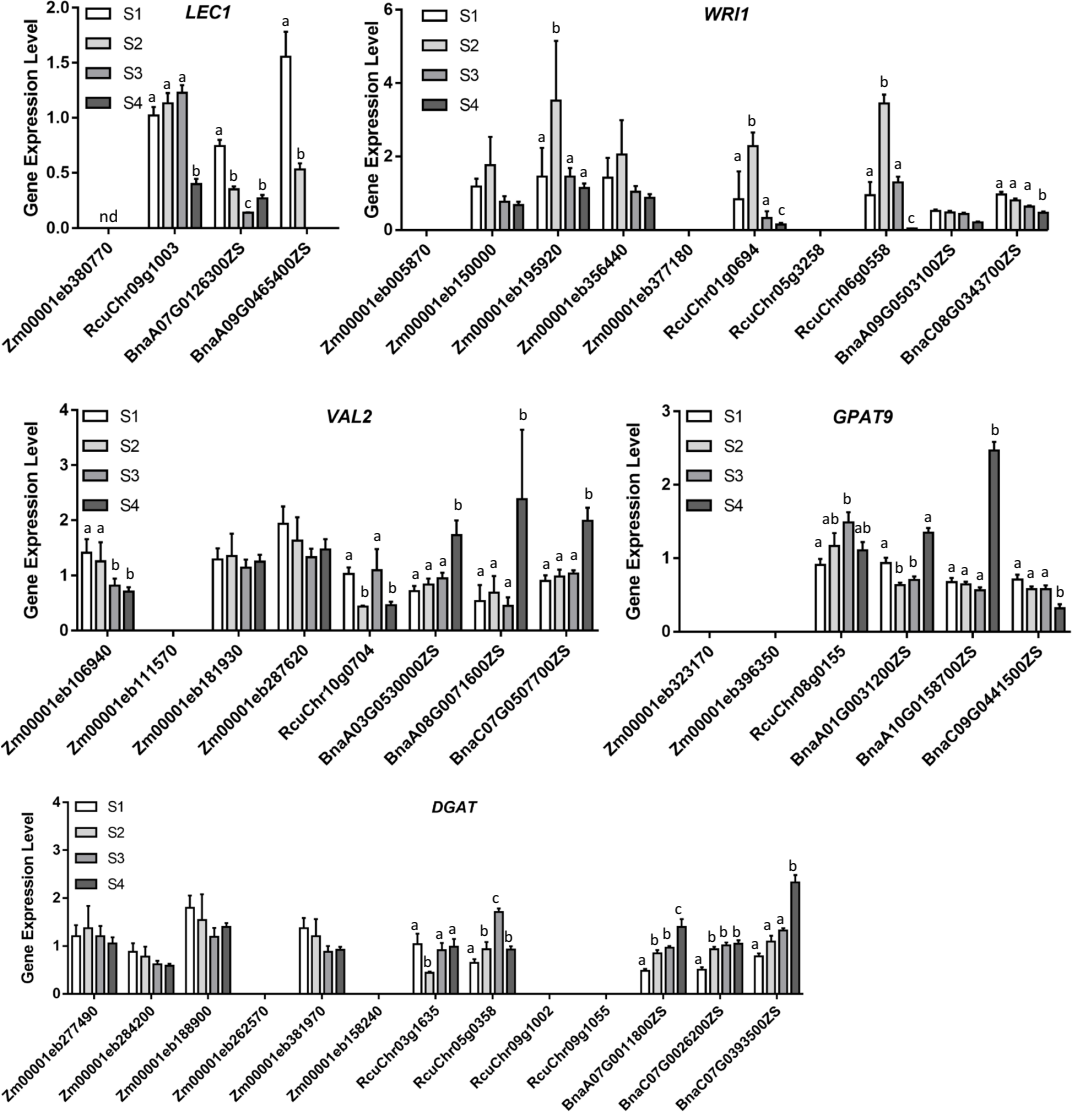
qRT-PCR verification of the expression of some vital genes (LEC1, WRI1, VAL2, GPAT9, and DGAT) in seed oil accumulation. The relative expression levels of LEC1, WRI1, VAL2, GPAT, and DGAT were quantified by qRT-PCR in maize, castor bean, and rapeseed. Actins were used as the internal control. Values are means with SD. One-way ANOVA was used to calculate significance for every gene

Next, the quantitative real-time PCR (qRT-PCR) were carried out to further validate the differential regulation of some critical genes (*WRI1*, *LEC1*, *VAL2*, *GPAT9*, and *DGAT*) that functioned in seed oil biosynthesis. In castor bean and rapeseed, the expression levels of *WRI1*, *LEC1*, *VAL2*, *GPAT9*, and *DGAT* genes increased or declined to two times between different developmental stages, which suggested that these genes were differentially expressed during the seed development (Fig. 6). On the contrary, the qRT-PCR analyses showed that the maize *LEC1* and *GPAT9* genes were not transcribed in maize embryo (Fig. 6). In addition, one out of four maize *VAL2* genes and two out of six maize *DGAT* genes were not transcribed in maize embryo, and expression levels of the remaining orthologous genes were not more than twice between four seed developmental stages (Fig. 6). These results implied that maize *LEC1*, *VAL2*, *GPAT9*, and *DGAT* genes were not differentially regulated during maize embryo development. Meantime, maize had five *WRI1s*, and the qRT-PCR data showed that two of them were not transcribed, two *WRI1s* were not differentially expressed, and one maize *WRI1* (*Zm00001eb195920*) were extremely highly expressed at stage 2 (Fig. 6), which is in line with that was found in comparative transcriptome analyses (Fig. S4). Thus, we concluded that *WRI1*, *LEC1*, *VAL2*, *GPAT9*, and *DGAT* genes were barely regulated in maize embryo development, which might account for the low oil content in maize seeds.

### Many PPP, the Calvin Cycle, and plastidic glycolytic genes that participated in carbohydrate metabolism were not regulated in maize embryo

**Fig. 7:**
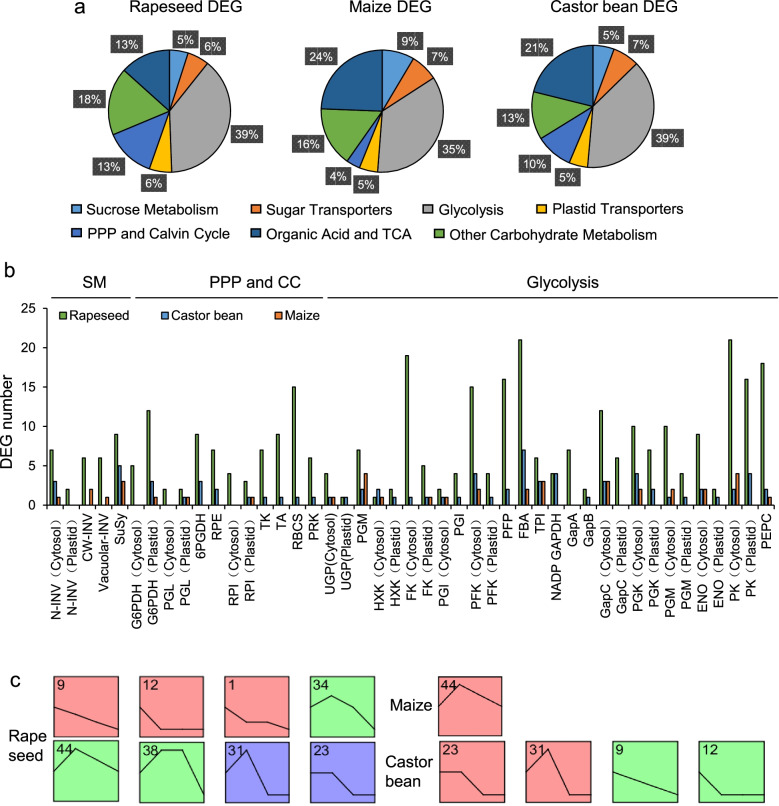
Differential regulation of CDEGs between three species. **a** pie chart showed the proportion of maize, castor bean, and rapeseed CDEGs in different carbon metabolism categories. **b** numbers of differential expressed genes that participated in different carbohydrate metabolism pathways such as sucrose metabolism (SM), glycolysis, pentose phosphate pathway and Calvin Cycle (PPP and CC) of maize, castor bean, and rapeseed during seed development. **c** the expression pattern clusters of carbohydrate-related genes in three plants

In addition to lipid-related genes, carbohydrate-related genes also play vital roles in seed oil biosynthesis. Comparison of carbohydrate-related metabolic pathways between species indicated that glycolysis, the PPP, and the Calvin Cycle were up-regulated in castor bean and rapeseed. Other carbohydrate metabolic pathways were up-regulated in castor bean and maize, while sucrose metabolism and sugar transport pathways were up-regulated in maize (Fig. 7a). The numbers of carbohydrate-related DEGs (CDEGs) associated with sugar transport, plastid transport, organic acids, the TCA, and other carbohydrate metabolic pathways were the highest in rapeseed, followed by castor bean and maize (Fig. S5).

Most enzymes that participate in the PPP and Calvin Cycle (e.g., 6-phosphogluconate dehydrogenase (6PGDH), ribulose-phosphate 3-epimerase (RPE), transketolase (TK), transaldolase (TA), ribulose bisphosphate carboxylase (RBCS), and phosphoribulokinase (PRK)) were not regulated in maize embryo (Fig. 7b). Numerous glycolytic enzymes (including key enzymes such as hexokinase (HXK), phosphofructokinase (PFK), phosphoglycerate kinase (PGK), and PK) were regulated only in the cytosol in maize embryo (Fig. 7b). By contrast, they were differentially expressed in the cytosol and plastid of rapeseed embryo and castor bean endosperm (Fig. 7b). More interestingly, *invertase* (*INV*) genes that play important roles in sucrose metabolism functioned only in the cytosol in castor bean endosperm. This differed from the situation in rapeseed and maize embryos, in which *INV* genes were differentially expressed in the cytosol, plastid, cell wall, and vacuole (Fig. 7b). Another vital sucrose metabolism enzyme, sucrose synthase (SuSy), plays important roles in carbon fixation and metabolism. We found that more *SuSy* homologs were regulated in castor bean and rapeseed than in maize: 9 of 14 *SuSy* genes were regulated in rapeseed, and all five *SuSy* genes were regulated in castor bean, but only 3 of 14 *SuSy* genes were regulated in maize (Fig. 1b and 7b). Analysis of gene expression patterns showed that most carbohydrate-related genes were highly expressed at stage 1 and stage 2 in castor bean and rapeseed, but were highly expressed at stage 2 and stage 3 in maize (Fig. 7c). We therefore speculated that reduced regulation of carbohydrate-related genes may limit the supply of substrates for oil biosynthesis in maize.

## Discussion

Thousands of genes were regulated during seed development, underscoring the sophisticated coordination of seed development and storage accumulation in plants. Plant seed oil biosynthesis is regulated by lipid- and carbohydrate-related genes [3]. Our research showed that most of these genes had the highest copy numbers in rapeseed, which is consistent with its allopolyploid genome in which most genes are duplicated and have redundant functions [55]. Most lipid- and carbohydrate-related genes had higher copy numbers in maize than in castor bean, which is reminiscent of the previous findings that key genes involved in oil biosynthesis and turnover have only single copies in castor bean [56]. Strikingly, more lipid- and carbohydrate-related genes were regulated during seed development in castor bean than in maize, and the proportion of regulated lipid- and carbohydrate-related genes was much higher in castor bean and rapeseed than in maize (Table 1 and Fig S1). These findings are in agreement with previous studies that acyl-lipid metabolism genes were differentially expressed between high-oil dicots and low-oil grasses [48]. Hence, we speculated that the lower oil content of maize embryo was not caused by gene family contraction but might owe to the less regulation of lipid- and carbohydrate-related genes.

Although the ratios of LDEG numbers to total DEG numbers were similar among three species, their gene expression patterns and lipid-related pathway enrichment were quite different. Fewer lipid biosynthesis pathways and more lipid metabolism pathways were regulated in maize, and lipid metabolism genes were down-regulated in castor bean and rapeseed but up-regulated in maize (Fig. 3). Therefore, we concluded that more lipid biosynthesis genes and fewer lipid metabolism genes were regulated during lipid accumulation in castor bean endosperm and rapeseed embryo than in maize embryo, potentially leading to the differences in seed oil accumulation between these species.

Broadly speaking, lipid biosynthesis in plants involves FA biosynthesis, TAG assembly, and oil body formation. Genes involved in TAG assembly and lipid-related transcription factor genes were minimally regulated in maize embryo, consistent with the GO enrichment analyses, which indicated that lipid biosynthesis pathways were specifically enriched only in castor bean and rapeseed (Fig. 4 and Table S2). Maize is one of the most important food crops and is increasingly viewed as a potential oil crop because of its high levels of polyunsaturated fatty acids [57, 58]. GWAS of 368 maize inbred lines showed that *DGAT1-2* and *WRI1a* contributed to the oil biosynthesis in Ilinois high-oil (IHO) population [51]. Similarly, the comparison between two contrasting maize genotypes revealed that many vital TAG biosynthetic genes showed differential expression between high oil content (HOC) and low oil content (LOC) maize. *GPAT*, *DGAT*, and *PDAT* genes were up-regulated, but *GPDH* was down-regulated in HOC [54]. These findings were consistent with our conclusion from comparisons across multiple species, which implied that *GPAT*, *DGAT*, *GPDH*, *PDAT*, and *WRI1* genes played vital roles in seed oil biosynthesis in maize kernels. Furthermore, *DGAT* gene, whose protein product catalyzes the last step in TAG formation, has been shown to play a vital role in maize seed oil biosynthesis. The oil and oleic acid contents increased by 41% and 107% when the high-oil *DGAT1-2* allele was ectopically expressed in maize kernels [59]. Likewise, heterologous expression of fungal *DGAT2* in maize caused a significant increase in the seed oil content [16]. Thus, we speculated that a lack of *DGAT* regulation was very likely to limit seed oil biosynthesis in maize. Transcription factors such as LEC1 and WRI1 have been proved to strongly influence seed oil synthesis in maize. Overexpression of *ZmLEC1* and *ZmWRI1* genes greatly increased the seed oil content of maize kernels and activated target genes that participate in lipid biosynthesis [23, 60]. Our research indicated that *LEC1* was not regulated in maize embryo, and only one out of five *WRI1* homologs was differentially expressed in this tissue. Thus, we inferred that the absence of regulation of lipid-related TFs and TAG assembly genes may also limit oil biosynthesis in maize embryo. These genes are therefore promising targets for the improvement of maize seed oil content.

Many carbohydrate-related genes were also differentially regulated in maize embryo. It has been reported that complete or partial glycolytic pathway can provide pyruvate for oil biosynthesis in non-photosynthetic plants [61]. Although cytosolic glycolysis is commonly regarded as the key carbon metabolism pathway in plants [62], plastidic glycolytic genes such as *PK* and *PGK* have also been shown to play important roles in carbon fixation, metabolism, and seed oil biosynthesis in Arabidopsis, rapeseed, and castor bean [28, 63–65]. However, according to our data, most glycolytic genes (e.g., *HXK*, *PFK*, *PGK*, and *PK*) were regulated specifically in the cytosol of maize. Moreover, most genes that participate in the PPP and the Calvin Cycle, which could provide substrates and energy for oil biosynthesis, were not regulated in maize embryo (Fig. 7). Thus, we speculated that levels of carbohydrates, which provide substrates for oil biosynthesis, might be limited in the maize embryo due to the absence of regulation of plastidic glycolytic, the PPP, and the Calvin Cycle genes.

Since copy numbers of lipid-related and carbohydrate-related genes were the lowest in castor bean among these species, an interesting question arises: how can castor bean rapidly accumulate so much lipids in seed? In vascular plants, sucrose is the main photoassimilate that moves from sources to sinks for the synthesis of storage compounds, and its metabolism is controlled by two enzymes, INV and SUSy [66]. In our study, only cytosol *INV* genes (which are minimally active in most plants) were differentially regulated in castor bean endosperm, whereas all castor bean *SUSy* genes were strongly regulated (Fig. 1b and 7b). These findings suggested that castor bean may prefer to utilize fructose for carbon fixation and metabolism. At the same time, lipid biosynthetic genes were highly expressed in castor bean (Fig. 5b), which might account in part for its high seed oil content. Besides, we speculated that castor bean may have evolved some new genes for lipid biosynthesis. This notion is consistent with previous research on the castor bean genome in which it was reported to have evolved an oleic acid hydroxylase gene (*FAH*) that functions in the biosynthesis of ricinoleic acid [56]. Therefore, it is probable that new genes have been evolved to maintain a high and specific flux of carbohydrates to TAGs in castor bean seeds, a hypothesis that will require experimental verification in the future.

## Conclusions

Transcriptomic comparisons between maize, castor bean, and rapeseed shed light on the gene regulation profiles of oil biosynthesis in oil storage tissues. The low oil content of maize embryo may have several causes: lower ratio of differentially expressed lipid- and carbohydrate-related genes; less regulation of lipid biosynthesis genes, especially TAG assembly genes and lipid-related TFs, but more regulation of lipid metabolism genes; and the shortage of substrates and energy for oil biosynthesis due to a lack of regulation of the PPP, the Calvin Cycle, and plastidic glycolytic genes. This study provides insight into molecular mechanisms of seed oil accumulation in maize and provides multiple potential targets for improving maize oil production, such as *DGAT*, *WRI1*, *LEC1*, *PK*, and *PGK*. These findings improve our understanding of seed oil biosynthesis at the molecular level and provide a solid foundation for further studies, helping to draw a blueprint for the molecular breeding of oilseed crops.

## Methods

### Plant growth conditions and sampling

The cultivated species of rapeseed (cv. ZS11), castor bean (cv. Youbi No. 4) and maize (cv. Taiyanghua No. 3) were used for analyses. All plants were cultivated at the experimental field with nature growth condition at Wuhan, Hubei, China. Rapeseed seeds were sampled at 15 (S1), 25 (S2), 35 (S3), and 50 (S4) days after flowering, castor bean seeds were sampled at 10 (S1), 20 (S2), 30 (S3), and 40 (S4) days after flowering, and maize seeds were sampled at 15 (S1), 20 (S2), 25 (S3), and 30 (S4) days after flowering according to the seed developmental process of plants. For each biological sample, developing seeds from four to six plants were collected and put together to do the analyses. Embryo of rapeseed and maize, and endosperm of castor bean at four stages were carefully separated from the whole seeds for oil content and transcriptome analysis.

### Analysis of SOC

The rape embryo, castor bean endosperm, and maize embryo with six biological replicates (for each biological sample, developing seeds from four to six plants were collected and put together to do the analyses) were dried at 37 °C to eliminate the moisture effect, since the developing maize, castor bean, and rapeseed seeds at pre-mature stage have lot of moisture. The seeds with known oil contents of each species were used to build the baseline for the measurement and the oil contents were measured using nuclear magnetic resonance (NMR PQ001; Niumag). The detection range of seed weight with NMR PQ001 was about 1.0–1.2 g.

### RNA extraction and cDNA synthesis

Total RNAs of rapeseed embryo, maize embryo, and castor bean endosperm were extracted for transcriptome analyses. Plant tissues were grounded with liquid nitrogen and total RNAs were isolated following manufacturer’s instructions of the RNA extraction kit (9769, TaKaRa). RNA concentration was measured using nanodrop spectrophotometer (Nanodrop Technologies, Santa Clara, CA, USA) and integrity was evaluated with the Agilent 2100 Bioanalyzer (Agilent Technologies, Santa Clara, CA, USA). Subsequently, an Illumina TruSeq RNA Sample Prep Kit (Illumina, San Diego, CA, USA) was used to build cDNA libraries.

### Transcriptome sequencing and blast searches

Generated cDNA libraries were sent to a HiSeq2500-PE125 platform (Illumina) to acquire sequence reads. The obtained raw data was processed with Trimmomatic [67] to filtered the adaptors and low-quality. Then, filtered clean reads were searched against the reference genome of *B. napus* (ZS11) [68], *R. communis* [69], and *Z. mays* (B73) from MaizeGDB (Assembly version: Zm-B73-REFERENCE-NAM-5.0), respectively. Gene expression levels were calculated by fragments per kilobase exon model per million mapped reads (FPKM) based on the number of uniquely mapped reads, to eliminate the influence of different gene lengths and sequencing discrepancies on the gene expression calculation. To identify DEGs during the seed development, S2, S3, and S4 samples were compared to S1 samples with DESeq2 [70] R package. The thresholds for significant differential expression were |log2 (Fold change)|≥ 1 and a corrected *p* < 0.05. *p*-values were adjusted using the Benjamini and Hochberg’s approach [71] for controlling the false discovery rate.

### Data analysis

The lipid-related the genes (genes associated with lipid metabolism and carbohydrate metabolism) which were used to carry out the genomic comparisons in this study referred to the published data in Plant Journal [47].

To analyze the copy number variation between maize, castor bean, and rapeseed, all genes of these three species were compared to Arabidopsis to find the homologous genes, and the copy numbers of the lipid-related genes were calculated in maize, castor bean, and rapeseed, respectively.

Gene expression pattern analyses were conducted with the STEM software, and the maximum number of model profiles were set to 50 and the maximum unit changes in model profiles between time points were set to 2. GO term enrichments were run on the RStudio loaded with the GO package, and the false discovery rate was set to 0.05. Venn diagram analyses were carried out using an online platform (http:// bioinfogp.cnb.csic.es/tools/venny/).

### Quantitative real-time PCR (qRT-PCR) analysis

Total RNAs were isolated from the embryos of maize and rapeseed, endosperm of castor bean at different developmental stages. cDNA was synthesized with 500 ng total RNA using PrimeScript® RT reagent Kit With gDNA Eraser (TaKaRa, Tokyo, Japan) according to manufacturer’s instructions. qRT-PCR was performed with iTaq Universal SYBR Green Supermix (Bio-Rad, USA). Gene transcript levels were quantified in triplicate by real-time PCR with the Applied Biosystems, 7500 Fast Real-Time PCR System (Thermo Fisher Scientific, USA). Six biological replicates were analyzed for each sample and the expression level was normalized to actin genes. The primer sequences used in this study are given in Table S3.

## Supplementary Information

**Additional file 1: Fig. S1. **The comparison of the regulated ratio of every homologous gene in the three species. R, rapeseed; C, castor bean; M, maize. **Fig. S2**. Gene expression patterns of all DEGs during seed development of rapeseed, castor, and maize. **Fig. S3**. Numbers of differentially expressed genes that participated in FA biosynthesis (a) and oil body formation (b) of rapeseed, castor bean, and maize during seed development. **Fig. S4**. The fold changes of key homologous lipid-related genes: FUS3, WRI1, LEC1/2, PDHC, GPDHC, DGAT. **Fig. S5**. Numbers of differential expressed genes that participated in sugar transporters (ST), plastid transporters (PT), organic acid and TCA (OA and TCA), and other carbohydrate metabolism (OCA) of rapeseed, castor bean, and maize during seed development.

**Additional  file 2: Table S1. **The list of the homologous lipid-related and carbohydrate-related genes in maize, castor bean, and rapeseed.  

**Additional  file 3: Table S2. **GO enrichment terms of total differential expressed maize, castor bean, and rapeseed genes, and the GO enrichment terms of conserved genes (shared by three species), dicot-specific (shared by castor bean and rapeseed) regulated genes, and embryo-specific (shared by rapeseed and maize) regulated genes.

**Additional file 4:Table S3.**Primer sequences for qRT-PCR verification in this study.

## Data Availability

All relevant data can be found within the article and its supporting information. The original sequencing data of transcriptomes are available at the NCBI under BioProjects PRJNA781731, PRJNA781735, and PRJNA781737.

## Abbreviations

SOC: Seed oil content
FA: Fatty acid
acetyl-CoA: Acetyl-Coenzyme A
ACCase: Acetyl-CoA carboxylase
MCMT: Malonyl-CoA:ACP malonyltransferase
KAS: β-Ketoacyl-ACP synthase
HAD: Hydroxyacyl-ACP dehydrase
ENR: Enoyl-ACP reductase
KAR: β-Ketoacyl-ACP reductase
ACP: Acyl carrier protein
SAD: Stearoyl-ACP desaturase
FAT: Acyl-ACP thioesterase
ER: Endoplasmic reticulum
TAG: Triacylglycerol
GPAT: Glyceral 3-phosphate acyltransferase
LPAAT: 1-Lysophosphatidic acid acyltransferase
PP: Phosphatidate phosphatase
DGAT: Diacylglycerol acyltransferase
PDAT: Phospholipid:diacylglycerol acyltransferase
TFs: Transcription factors
WRI1: WRINKLED1
LEC1/2: LEAFY COTYLEDON1/2
ABI3: ABSCISIC ACID INSENSITIVE3
FUS3: FUSCA3
PKL: PICKLE
VAL: VP1/ABI3-LIKE
PPP: Pentose phosphate pathway
PK: Pyruvate kinase
PEPCase: Phosphoenolpyruvate carboxylase
OAA: Oxaloacetate
QTL: Quantitative trait locus
GWAS: Genome-wide association study
EST: Expressed sequence tags
GO: Gene Ontology
LDEGs: Lipid-related differentially expressed genes
GPDH: Glyceraldehyde-3-phosphate dehydrogenase
qRT-PCR: Quantitative real-time PCR
CDEGs: Carbohydrate-related differentially expressed genes
6PGDH: 6-Phosphogluconate dehydrogenase
RPE: Ribulose-phosphate 3-epimerase
TK: Transketolase
TA: Transaldolase
RBCS: Ribulose bisphosphate carboxylase
PRK: Phosphoribulokinase
HXK: Hexokinase
PFK: Phosphofructokinase
PGK: Phosphoglycerate kinase
INV: Invertase
SuSy: Sucrose synthase
IHO: Llinois high-oil
HOC: High oil content
LOC: Low oil content
FPKM: Fragments per kilobase exon model per million mapped reads

## References

[1] Jaworski J, Cahoon EB (2003). Industrial oils from transgenic plants. Curr Opin Plant Biol.

[2] Durrett TP, Benning C, Ohlrogge J (2008). Plant triacylglycerols as feedstocks for the production of biofuels. Plant J.

[3] Li-Beisson Y, Shorrosh B, Beisson F, Andersson MX, Arondel V, Bates PD (2010). Acyl-lipid metabolism. Arabidopsis Book..

[4] Cui YP, Liu ZJ, Zhao YP, Wang YM, Huang Y, Li L (2017). Overexpression of Heteromeric *GhACCase* Subunits Enhanced Oil Accumulation in Upland Cotton. Plant Mol Biol Rep.

[5] Voelker TA, Hayes TR, Cranmer AM, Turner JC, Davies HM (1996). Genetic engineering of a quantitative trait: Metabolic and genetic parameters influencing the accumulation of laurate in rapeseed. Plant J.

[6] Roesler K, Shintani D, Savage L, Boddupalli S, Ohlrogge J (1997). Targeting of the Arabidopsis homomeric acetyl-coenzyme A carboxylase to plastids of rapeseeds. Plant Physiol.

[7] Stoll C, Luhs W, Zarhloul MK, Brummel M, Spener F, Friedt W (2006). Knockout of KASIII regulation changes fatty acid composition in canola (*Brassica napus*). Eur J Lipid Sci Tech.

[8] Wei Q, Li J, Zhang L, Wu PZ, Chen YP, Li MR (2012). Cloning and characterization of a beta-ketoacyl-acyl carrier protein synthase II from *Jatropha curcas*. J Plant Physiol.

[9] Bourgis F, Kilaru A, Cao X, Ngando-Ebongue GF, Drira N, Ohlrogge JB (2011). Comparative transcriptome and metabolite analysis of oil palm and date palm mesocarp that differ dramatically in carbon partitioning. Proc Natl Acad Sci USA.

[10] Salas JJ, Ohlrogge JB (2002). Characterization of substrate specificity of plant FatA and FatB acyl-ACP thioesterases. Arch Biochem Biophys.

[11] Shockey J, Regmi A, Cotton K, Adhikari N, Browse J, Bates PD (2016). Identification of Arabidopsis *GPAT9* (*At5g60620*) as an Essential Gene Involved in Triacylglycerol Biosynthesis. Plant Physiol.

[12] Jain RK, Coffey M, Lai K, Kumar A, MacKenzie SL (2000). Enhancement of seed oil content by expression of glycerol-3-phosphate acyltransferase genes. Biochem Soc T.

[13] Zou JT, Katavic V, Giblin EM, Barton DL, MacKenzie SL, Keller WA (1997). Modification of seed oil content and acyl composition in the brassicaceae by expression of a yeast *sn*-2 acyltransferase gene. Plant Cell.

[14] Baud S, Lepiniec L (2010). Physiological and developmental regulation of seed oil production. Prog Lipid Res.

[15] Jako C, Kumar A, Wei YD, Zou JT, Barton DL, Giblin EM (2001). Seed-specific over-expression of an Arabidopsis cDNA encoding a diacylglycerol acyltransferase enhances seed oil content and seed weight. Plant Physiol.

[16] Oakes J, Brackenridge D, Colletti R, Daley M, Hawkins DJ, Xiong H (2011). Expression of fungal *diacylglycerol acyltransferase2* genes to increase kernel oil in maize. Plant Physiol.

[17] Lardizabal K, Effertz R, Levering C, Mai J, Pedroso MC, Jury T (2008). Expression of *Umbelopsis ramanniana DGAT2A* in seed increases oil in soybean. Plant Physiol.

[18] Dahlqvist A, Stahl U, Lenman M, Banas A, Lee M, Sandager L (2000). Phospholipid : diacylglycerol acyltransferase: An enzyme that catalyzes the acyl-CoA-independent formation of triacylglycerol in yeast and plants. Proc Natl Acad Sci USA.

[19] Stahl U, Carlsson AS, Lenman M, Dahlqvist A, Huang BQ, Banas W (2004). Cloning and functional characterization of a Phospholipid : Diacylglycerol acyltransferase from Arabidopsis. Plant Physiol.

[20] Siloto RMP, Findlay K, Lopez-Villalobos A, Yeung EC, Nykiforuk CL, Moloney MM (2006). The accumulation of oleosins determines the size of seed oilbodies in *Arabidopsis*. Plant Cell.

[21] Grimberg A, Wilkinson M, Snell P, De Vos RP, Gonzalez-Thuillier I, Tawfike A (2020). Transitions in wheat endosperm metabolism upon transcriptional induction of oil accumulation by oat endosperm WRINKLED1. BMC Plant Biol.

[22] Chen B, Zhang G, Li P, Yang J, Guo L, Benning C (2020). Multiple GmWRI1s are redundantly involved in seed filling and nodulation by regulating plastidic glycolysis, lipid biosynthesis and hormone signalling in soybean (*Glycine max*). Plant Biotechnol J.

[23] Shen B, Allen WB, Zheng P, Li C, Glassman K, Ranch J (2010). Expression of *ZmLEC1* and *ZmWRI1* increases seed oil production in maize. Plant Physiol.

[24] Elahi N, Duncan RW, Stasolla C (2015). Decreased seed oil production in *FUSCA3 Brassica napus* mutant plants. Plant Physiol Bioch.

[25] Schwender J, Ohlrogge JB (2002). Probing in vivo metabolism by stable isotope labeling of storage lipids and proteins in developing *Brassica napus* embryos. Plant Physiol.

[26] Kubis SE,  Pike  MJ, Hill LM, Rawsthorne S (2004). The import of phosphoenolpyruvate by plastids from developing embryos of oilseed rape, *Brassica napus* (L.), and its potential as a substrate for fatty acid synthesis. J Exp Bot.

[27] Schwender J, Ohlrogge JB, Shachar-Hill Y (2003). A flux model of glycolysis and the oxidative pentosephosphate pathway in developing *Brassica napus* embryos. J Biol Chem.

[28] Andre C, Froehlich JE, Moll MR, Benning C (2007). A heteromeric plastidic pyruvate kinase complex involved in seed oil biosynthesis in *Arabidopsis*. Plant Cell.

[29] Baud S, Wuilleme S, Dubreucq B, de Almeida A, Vuagnat C, Lepiniec L (2007). Function of plastidial pyruvate kinases in seeds of *Arabidopsis thaliana*. Plant J.

[30] Xu ZP, Li JW, Guo XP, Jin SX, Zhang XL (2016). Metabolic engineering of cottonseed oil biosynthesis pathway via RNA interference. Sci Rep..

[31] Lu L, We W, Li QT, Bian  XH, Lu X, Hu  Y  (2021). A transcriptional regulatory module controls lipid accumulation in soybean. New Phytol.

[32] Wang LH, Zhang YX, Li DH, Dossa K, Wang ML, Zhou R (2019). Gene expression profiles that shape high and low oil content sesames. BMC Genet..

[33] Liu S, Fan CC, Li JN, Cai GQ, Yang QY, Wu J (2016). A genome-wide association study reveals novel elite allelic variations in seed oil content of *Brassica napus*. Theor Appl Genet.

[34] Wang L, Jiang X, Wang L, Wang W, Fu C, Yan X (2019). A survey of transcriptome complexity using PacBio single-molecule real-time analysis combined with Illumina RNA sequencing for a better understanding of ricinoleic acid biosynthesis in *Ricinus communis*. BMC Genomics.

[35] Cocuron JC, Koubaa M, Kimmelfield R, Ross Z, Alonso AP (2019). A Combined Metabolomics and Fluxomics Analysis Identifies Steps Limiting Oil Synthesis in Maize Embryos. Plant Physiol.

[36] Hayden DM, Rolletschek H, Borisjuk L, Corwin J, Kliebenstein DJ, Grimberg A (2011). Cofactome analyses reveal enhanced flux of carbon into oil for potential biofuel production. Plant J.

[37] Guerin C, Joet T, Serret J, Lashermes P, Vaissayre V, Agbessi MD (2016). Gene coexpression network analysis of oil biosynthesis in an interspecific backcross of oil palm. Plant J.

[38] Cao YC, Li SG, Wang ZL, Chang FG, Kong JJ, Gai JY (2017). Identification of Major Quantitative Trait Loci for Seed Oil Content in Soybeans by Combining Linkage and Genome-Wide Association Mapping. Front Plant Sci..

[39] Chen G, Geng JF, Rahman M, Liu XP, Tu JX, Fu TD (2010). Identification of QTL for oil content, seed yield, and flowering time in oilseed rape (*Brassica napus*). Euphytica.

[40] Jiang  CC , Shi JQ,, Li RY, Long Y, Wang H, Li  DR (2014). Quantitative trait loci that control the oil content variation of rapeseed (*Brassica napus* L.). Theor Appl Genet.

[41] Osorio-Guarin JA, Garzon-Martinez GA, Delgadillo-Duran P, Bastidas S, Moreno LP, Enciso-Rodriguez FE (2019). Genome-wide association study (GWAS) for morphological and yield-related traits in an oil palm hybrid (*Elaeis oleifera* x *Elaeis guineensis*) population. BMC Plant Biol.

[42] Hwang EY, Song QJ, Jia GF, Specht JE, Hyten DL, Costa J (2014). A genome-wide association study of seed protein and oil content in soybean. BMC Genomics..

[43] Tang S, Zhao H, Lu SP, Yu LQ, Zhang GF, Zhang YT (2021). Genome- and transcriptome-wide association studies provide insights into the genetic basis of natural variation of seed oil content in *Brassica napus*. Mol Plant.

[44] Dussert S, Guerin C, Andersson M, Joet T, Tranbarger TJ, Pizot M (2013). Comparative transcriptome analysis of three oil palm fruit and seed tissues that differ in oil content and fatty acid composition. Plant Physiol.

[45] Huang J, Zhang T, Zhang Q, Chen M, Wang Z, Zheng B (2016). The mechanism of high contents of oil and oleic acid revealed by transcriptomic and lipidomic analysis during embryogenesis in *Carya cathayensis* Sarg. BMC Genomics.

[46] Liu Q, Sun Y, Chen J, Li P, Li C, Niu G (2016). Transcriptome analysis revealed the dynamic oil accumulation in *Symplocos paniculata* fruit. BMC Genomics.

[47] Troncoso-Ponce MA, Kilaru A, Cao X, Durrett TP, Fan J, Jensen JK (2011). Comparative deep transcriptional profiling of four developing oilseeds. Plant J.

[48] Zhang L, Wang SB, Li QG, Song J, Hao YQ, Zhou L (2016). An integrated bioinformatics analysis reveals divergent evolutionary pattern of oil biosynthesis in high- and low-oil plants. PLoS ONE..

[49] Zhang Z, Dunwell JM, Zhang YM (2018). An integrated omics analysis reveals molecular mechanisms that are associated with differences in seed oil content between *Glycine max* and *Brassica napus*. BMC Plant Biol.

[50] Zhou H, Xia D, Li P, Ao Y, Xu X, Wan S et al. Genetic architecture and key genes controlling the diversity of oil composition in rice grains. Mol Plant. 2020. 10.1016/j.molp.2020.12.001 33307246

[51] Li H, Peng Z, Yang X, Wang W, Fu J, Wang J (2013). Genome-wide association study dissects the genetic architecture of oil biosynthesis in maize kernels. Nat Genet.

[52] Cagliari A, Margis-Pinheiro M, Loss G, Mastroberti AA, de Araujo Mariath JE, Margis R (2010). Identification and expression analysis of castor bean (*Ricinus communis*) genes encoding enzymes from the triacylglycerol biosynthesis pathway. Plant Sci.

[53] Amiruddin N, Chan PL, Azizi N, Morris PE, Chan KL, Ong PW (2020). Characterization of Oil Palm Acyl-CoA-Binding Proteins and Correlation of Their Gene Expression with Oil Synthesis. Plant Cell Physiol.

[54] Zhang X, Hong M, Wan H, Luo L, Yu Z, Guo R (2019). Identification of Key Genes Involved in Embryo Development and Differential Oil Accumulation in Two Contrasting Maize Genotypes. Genes..

[55] Chalhoub B, Denoeud F, Liu SY, Parkin IAP, Tang HB, Wang XY (2014). Early allopolyploid evolution in the post-Neolithic *Brassica napus* oilseed genome. Science.

[56] Chan AP, Crabtree J, Zhao Q, Lorenzi H, Orvis J, Puiu D (2010). Draft genome sequence of the oilseed species *Ricinus communis*. Nat Biotechnol.

[57] Godfray HCJ, Beddington JR, Crute IR, Haddad L, Lawrence D, Muir JF (2010). Food Security: The Challenge of Feeding 9 Billion People. Science.

[58] Chen J, Zeng B, Zhang M, Xie SJ, Wang GK, Hauck A (2014). Dynamic Transcriptome Landscape of Maize Embryo and Endosperm Development. Plant Physiol.

[59] Zheng P, Allen WB, Roesler K, Williams ME, Zhang S, Li J (2008). A phenylalanine in DGAT is a key determinant of oil content and composition in maize. Nat Genet.

[60] Pouvreau B, Baud S, Vernoud V, Morin V, Py C, Gendrot G (2011). Duplicate maize *Wrinkled1* transcription factors activate target genes involved in seed oil biosynthesis. Plant Physiol.

[61] Neuhaus HE, Emes MJ (2000). Nonphotosynthetic metabolism in plastids. Annu Rev Plant Phys.

[62] Rawsthorne S (2002). Carbon flux and fatty acid synthesis in plants. Prog Lipid Res.

[63] Li RZ, Qiu ZM, Wang XG, Gong PP, Xu QZ, Yu QB (2019). Pooled CRISPR/Cas9 reveals redundant roles of plastidial phosphoglycerate kinases in carbon fixation and metabolism. Plant J.

[64] White JA, Todd  T, Newman T, Focks N, Girke T, de Ilarduya  OM (2000). A new set of Arabidopsis expressed sequence tags from developing seeds. The metabolic pathway from carbohydrates to seed oil. Plant Physiol.

[65] Simcox PD, Reid EE, Canvin DT, Dennis DT (1977). Enzymes of the Glycolytic and Pentose Phosphate Pathways in Proplastids from the Developing Endosperm of *Ricinus communis* L. Plant Physiol.

[66] Koch K (2004). Sucrose metabolism: regulatory mechanisms and pivotal roles in sugar sensing and plant development. Curr Opin Plant Biol.

[67] Bolger AM, Lohse M, Usadel B (2014). Trimmomatic: a flexible trimmer for Illumina sequence data. Bioinformatics.

[68] Song JM, Guan ZL, Hu JL, Guo CC, Yang ZQ, Wang S (2020). Eight high-quality genomes reveal pan-genome architecture and ecotype differentiation of Brassica napus. Nat Plants.

[69] Lu J, Pan C, Fan W, Liu W, Zhao H, Li D et al. A Chromosome-level Assembly of A Wild Castor Genome Provides New Insights into the Adaptive Evolution in A Tropical Desert. Genomics, Proteomics & Bioinformatics. 2021; doi:10.1016/j.gpb.2021.04.003 10.1016/j.gpb.2021.04.003 PMC9510866 34339842

[70] Love MI, Huber W, Anders S (2014). Moderated estimation of fold change and dispersion for RNA-seq data with DESeq2. Genome Biol.

[71] Benjamini Y, Yekutieli D (2001). The control of the false discovery rate in multiple testing under dependency. Ann Stat.

