# Supplementary material for: An integrated omics analysis reveals the gene expression profiles of maize, castor bean, and rapeseed for seed oil biosynthesis
Source: BMC Plant Biol. 2022 Mar 29;22:153. doi: 10.1186/s12870-022-03495-y (PMC8966334; doi:10.1186/s12870-022-03495-y)
Supplement: Supplementary file 1 — Additional file 1: Fig. S1. The comparison of the regulated ratio of every homologous gene in the three species. R, rapeseed; C, castor bean; M, maize. Fig. S2. Gene expression patterns of all DEGs during seed development of rapeseed, castor, and maize. Fig. S3. Numbers of differentially expressed genes that participated in FA biosynthesis (a) and oil body formation (b) of rapeseed, castor bean, and maize during seed development. Fig. S4. The fold changes of key homologous lipid-related genes: FUS3, WRI1, LEC1/2, PDHC, GPDHC, DGAT. Fig. S5. Numbers of differential expressed genes that participated in sugar transporters (ST), plastid transporters (PT), organic acid and TCA (OA and TCA), and other carbohydrate metabolism (OCA) of rapeseed, castor bean, and maize during seed development. [file 12870_2022_3495_MOESM1_ESM.pdf]

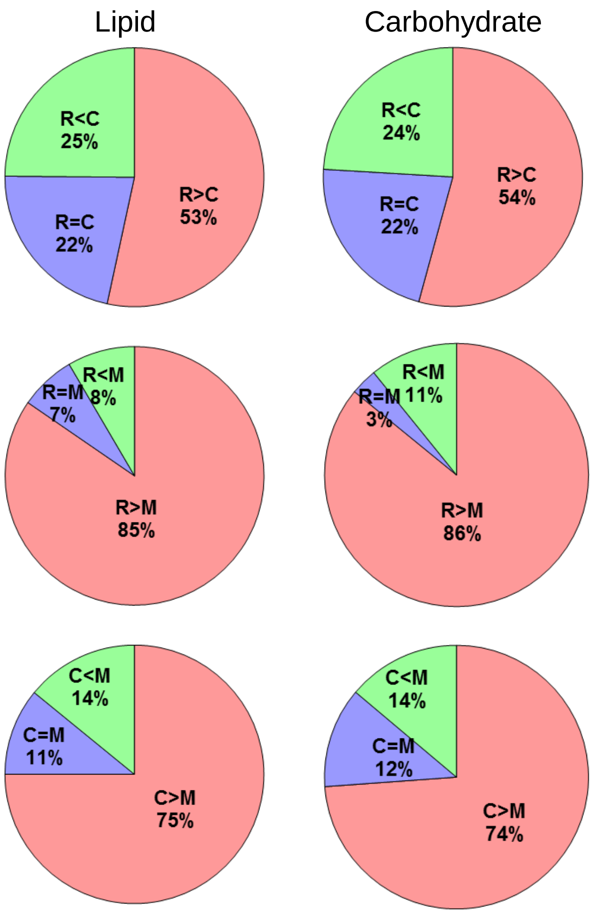

Fig. S1. The comparison of the regulated ratio of every homologous gene in the three species. R, rapeseed; C, castor bean; M, maize.

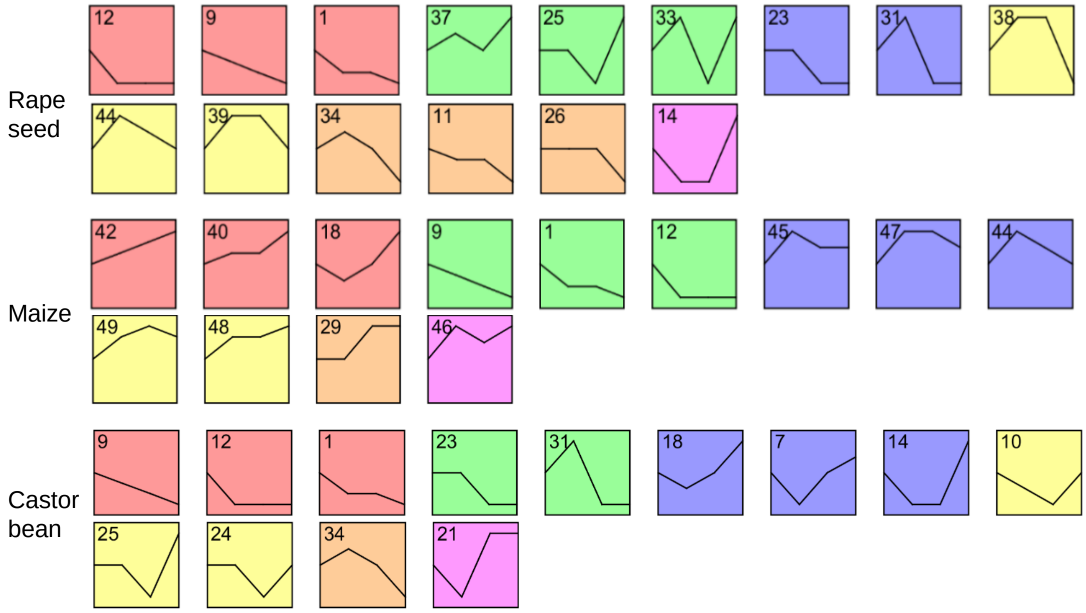

Fig. S2. Gene expression patterns of all DEGs during seed development of rapeseed, castor, and maize.

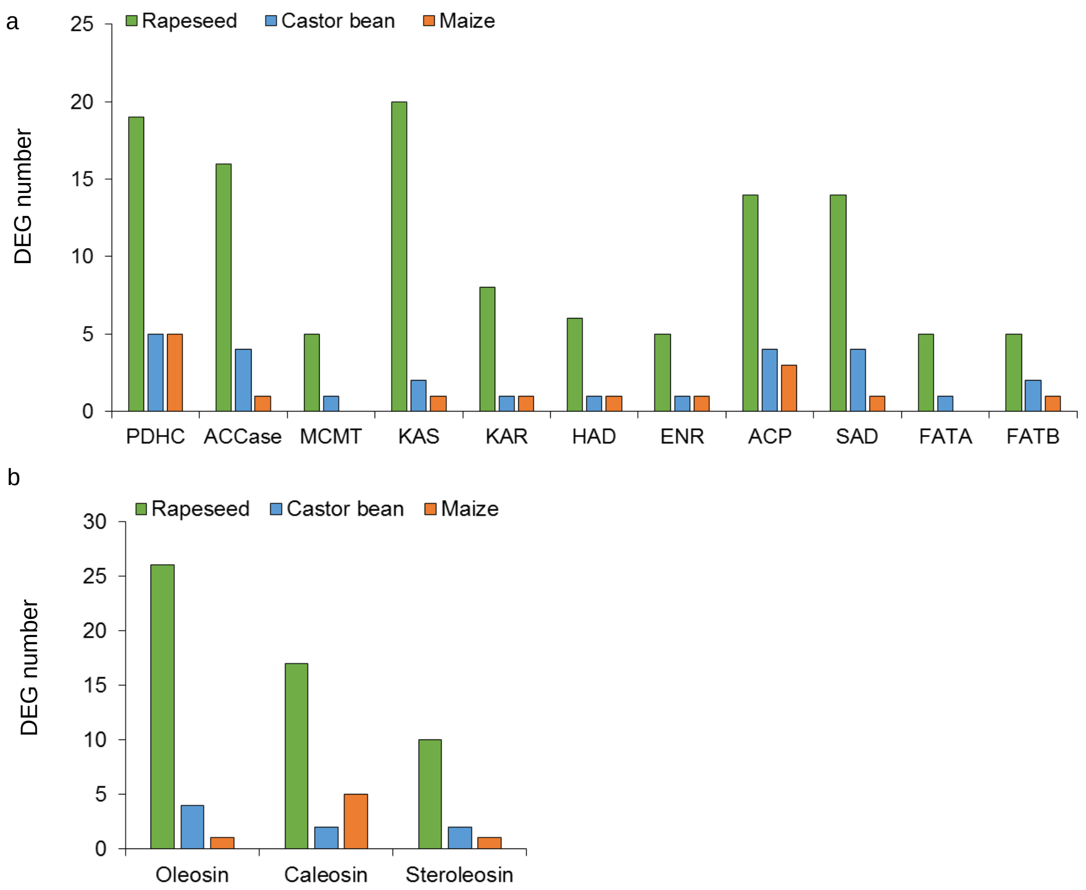

Fig. S3. Numbers of differentially expressed genes that participated in FA biosynthesis (**a**) and oil body formation (**b**) of rapeseed, castor bean, and maize during seed development.

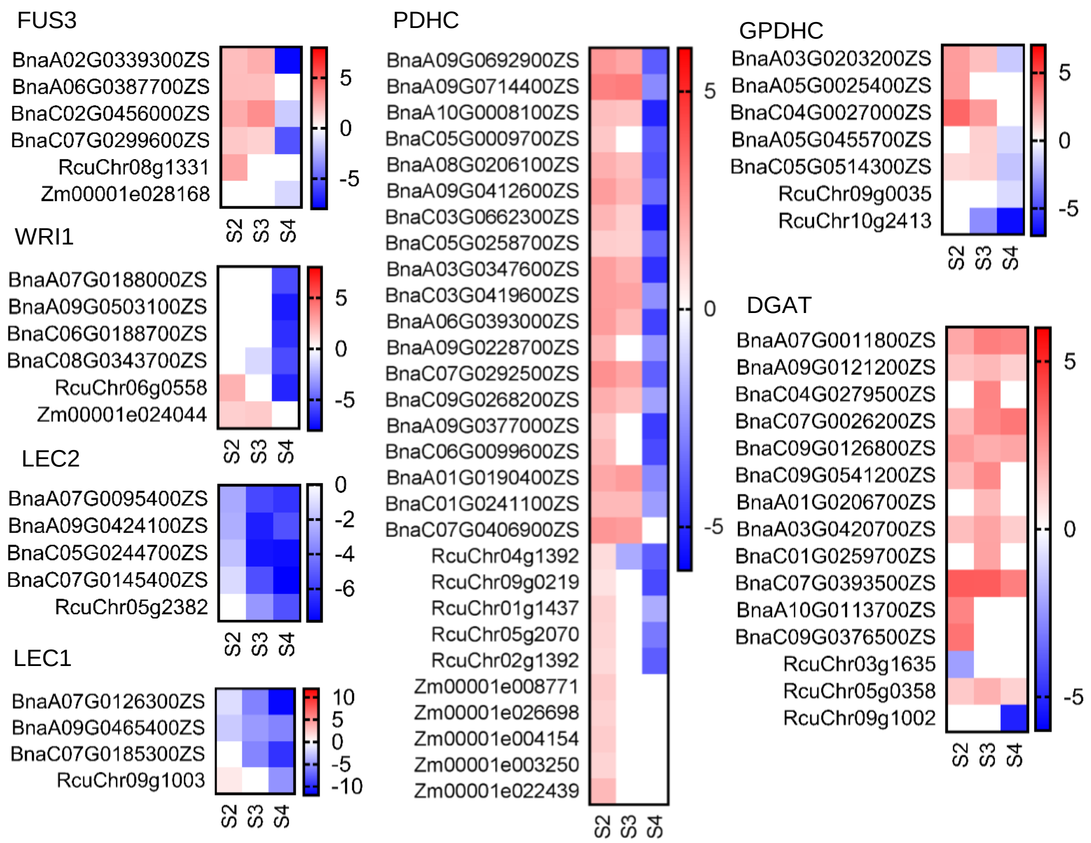

Fig. S4. The fold changes of key homologous lipid-related genes: *FUS3*, *WRI1*, *LEC1/2*, *PDHC*, *GPDHC*, *DGAT*.

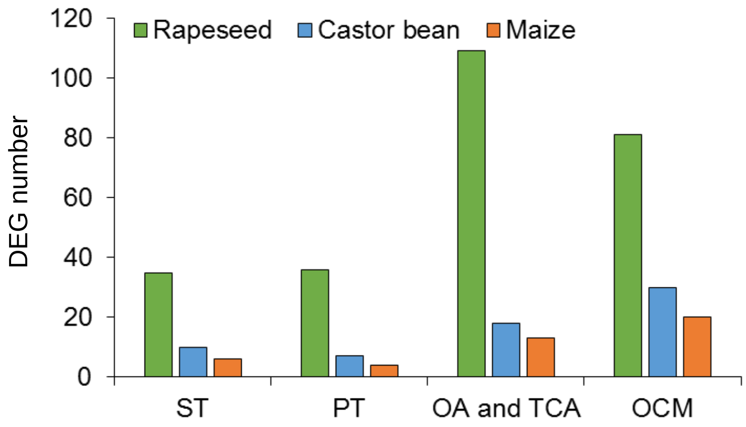

Fig. S5. Numbers of differential expressed genes that participated in sugar transporters (ST), plastid transporters (PT), organic acid and TCA (OA and TCA), and other carbohydrate metabolism (OCA) of rapeseed, castor bean, and maize during seed development.
